# Supplementary material for: Magnesium Isoglycyrrhizinate Reduces the Target-Binding Amount of Cisplatin to Mitochondrial DNA and Renal Injury through SIRT3
Source: Int J Mol Sci. 2022 Oct 28;23(21):13093. doi: 10.3390/ijms232113093 (PMC9654254; doi:10.3390/ijms232113093)
Supplement: Supplementary file 1 [file ijms-23-13093-s001.zip › ijms-1923377-supplementary.pdf]

Supplement Figure S1

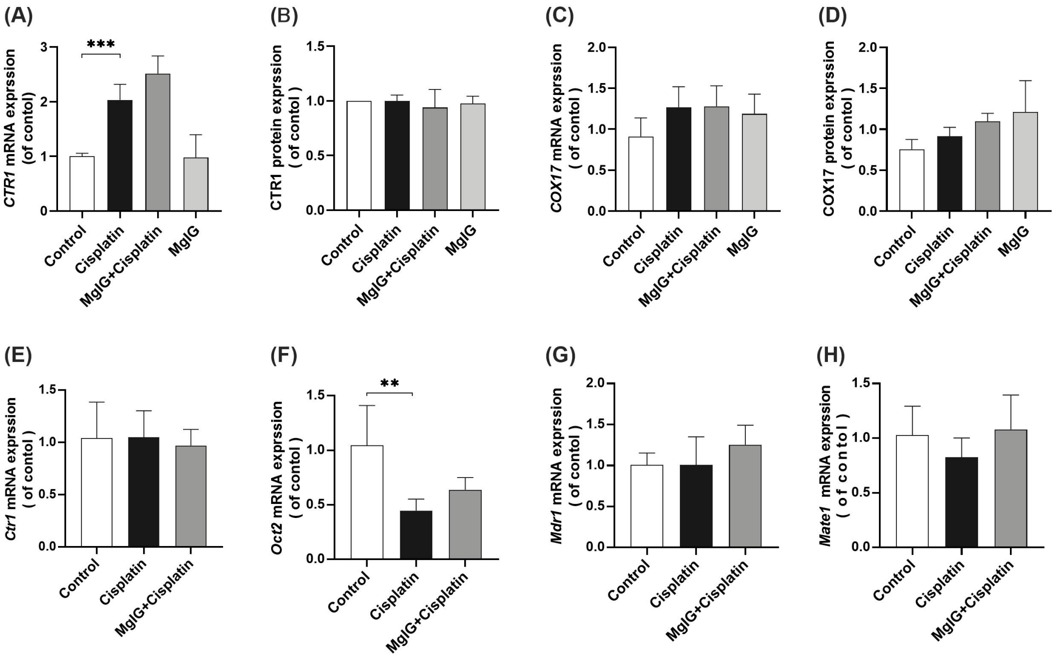

The effect of MgIG on cisplatin-associated transporters in HK-2 cells. A-B The mRNA and protein expression of CTR1 in HK-2 cells. **C-D** The mRNA and protein expression of COX17 in HK-2 cells. MgIG has no effect on the mRNA and protein expression of CTR1 and COX17 in HK-2 cells. **E-H** The mRNA expression of *Ctr1*， *Oct2*, *Mdr1*, *Mate1* in mice kidneys. MgIG has no effect on the mRNA expression of *Ctr1*， *Oct2*, *Mdr1*, *Mate1* in mice kidneys. The data are presented as the mean ± SD, n = 6. ** $P < 0.01$, *** $P < 0.001$.

Supplement Figure S2

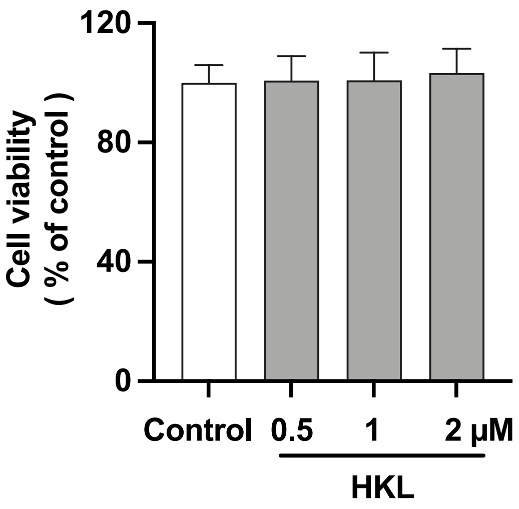

The effect of HKL on cell viability of HK-2 cells. With co-administration of HKL at 0.5, 1, and 2 μM for 48h, the cell viability appeared no change. The data are presented as the mean ± SD, n = 6.

Supplement Table S1

Table S1 Detective parameters

Compound	Detection mode	DP	CE	Quantitative ion pairs	Retention time
NAM	+	60	25	123.3 / 80.0	2.13
NR	+	40	17	255.2 / 123.1	3.04
NMN	+	50	20	335.2 / 123.0	7.44
NADPH	-	-190	-50	744.0 / 408.0	6.66

Supplement Table S2

Table S2. The primer sequences of RT-PCR.

	Forward Primer (5'-3')	Reverse Primer (5'-3')
Mus *Actb*	ACGGCCAGGTCATCACTATTG	AGGGGCCGGACTCATCGTA
Mus *Kim-1*	AGCAGTCGGTACAACTTAAAGG	ACTCGACAACAATACAGACCAC
Mus *Ngal*	TGGCCCTGAGTGTCATGTG	CTCTTGTAGCTCATAGATGGTGC
Mus *Ctr1*	CATGATGATGATGCCTATGACC	CAGCATCTGCTGCCCAAC
Mus *Oct2*	GGGTTCTCATGGCGATTTCAC	GCCCAGCCCAACAAATTCTG
Mus *Mdr1*	TCGACCCACACTTCAGCTACA	AGCCGGTTGTCCATGTTGG
Mus *Mate1*	GTTGGCCTTACGGAGAGGAC	AATCCCACCCACCAAGACTAA
Homo *ACTB*	ACGGCCAGGTCATCACTATTG	AGGGGCCGGACTCATCGTA
Homo *SIRT3*	GGCTCTATACACAGAACATCGAC	TAGCTGTTACAAAGGTCCCGT
Homo *CTR1*	AAGGACTCAAGATAGCCCGAG	TGGGACAGGCATGGAATTGTA
Homo *COX17*	GGCTCTATACACAGAACATCGAC	TAGCTGTTACAAAGGTCCCGT
